# Plunder of Human Blood Leukocytes Containing Ingested Material, by Other Leukocytes: Where Is the Fusagen That Allows Preservation of Membrane Integrity and Motile Function?

**DOI:** 10.1371/journal.pone.0065796

**Published:** 2013-06-26

**Authors:** Stephen E. Malawista, Anne Chevance de Boisfleury

**Affiliations:** 1 Department of Internal Medicine, Yale University School of Medicine, New Haven, Connecticut, United States of America; 2 Centre d'Ecologie Cellulaire, Hôpital de la Salpétrière, Paris, France; Federal Institute for Vaccines and Biomedicines, Germany

## Abstract

In studying phagocytosis of zymosan particles by human blood monocytes in phase-contrast videomicroscopy, we found that monocytes loaded with zymosan particles became chemotactic for polymorphonuclear leukocytes (PMN) which closed on them and purloined their particle content. This despoliation usually occurred in monocytes that had begun to swell—prefiguring their death. The violent seizure of their contents by the aggressing PMN often tore the monocytes apart. However, some apparently healthy monocyte survived the removal of zymosan content by PMN or, more commonly, its removal by another monocyte. PMN—a much hardier cell in slide preparations—that were similarly loaded with zymosan particles, also attracted PMN. The latter could remove zymosan from the target cell without killing it. Thus, leukocytes were sacrificing significant portions of themselves without losing residual membrane integrity and motile function. Their behavior with respect to other particles (e.g., bacteria) will be of interest. We suggest that the membrane fusagen resides in the inner membrane leaflets when they are brought together in an extreme hourglass configuration. This event may be similar to the fragmentation of erythrocytes into intact pieces, the formation of cytokineplasts, the rear extrusion of content by migrating cells on surfaces, and the phagocytic process itself.

## Introduction

In studying phagocytosis of zymosan particles by human blood monocytes in phase-contrast videomicroscopy, we came upon a phenomenon previously unreported: monocytes loaded with zymosan particles can attract PMN which can ingest the monocyte's already ingested contents. Our purpose here is to describe this process as observed in phase-contrast videomicroscopy and to speculate on how it comes about.

## Materials and Methods

### Ethics Statement

Anonymous blood donors were obtained with informed consent from ‘Le Don du Sang à Paris” (blood bank).

### Zymosan

We suspended Zymosan A (Sigma Z 4250) 1 mg/ml PBS under sterile conditions, and washed it a second time in the media to be used.

### Leukocytes

We collected fresh, heparinized (with sodium heparin) blood in two or three 7-ml tubes from anonymous human donors and allowed them to sediment at an angle of 60° at room temperature for 1 h. We concentrated leukocytes from the buffy coat in a microcentrifuge (Costar, Cambridge, MA, USA; Model #8455) for 30 s at 10,000 rpm (5585 g), resuspended them three times in autologous platelet-poor plasma, sedimenting them each time at 1,000 rpm for 1 min to remove most residual platelets, and then resuspended them in 1 ml of plasma or of autologous serum. We separated monocytes and neutrophils by sedimentation in a separation medium (CMSMSLO1-OU, Eurobio), and washed the cells twice in designated media, made to 100 µl. For studies of decomplemented serum we used 10% decomplemented (56°C for 30 min.) autologous serum.

### Zymosan-leukocyte interactions

To the sediment from 25 µl of the zymosan suspension, we added 50 µl of cells (monocytes or neutrophils), resuspended the mixture, and incubated it at 37°C for 1–2 hr (monocytes) or 30 min (neutrophils). After this period of phagocytosis we added remaining neutrophils or monocytes in high concentration, from the bottom of their tube where they had been lightly sedimented. We quickly deposited a drop of this suspension, sufficient to wet an entire overlying, 22-mm×32-mm coverslip (∼4 µl) on a clean glass slide precoated, when monocytes were employed, with glycol methacrylate (29) or on a plastic slide, sealed the preparation with paraffin, and removed it to the warmed (33°C) stage of a Zeiss phase-contrast photomicroscope (objective, 40×), which was connected via a Hamamatsu microscope video camera C2400 (Hamamatsu Photonics K. K., Hamamatsu City, Japan) to a Panasonic time-lapse video recorder AG6720 (Matsushita Electric Industrial, Osaka, Japan) or, later, via a video camera (PL-E421MU-Kit, PixeLINK, Ottowa, CA) to a computer. Zymosan appears as round, 3–4 µm particles with punctate black centers. Videos are ∼15× real time.

## Results

Monocytes are fragile in our sealed slide preparations. On glass they displace themselves little, and generally last no more than several minutes before they begin to swell or become mummified. They move more freely on methacrylate-coated glass or on plastic, and may survive for an hour or two. Our final media was 100% autologous plasma except as noted; 100% autologous serum and 10% of either one in buffer were also useful.

**Figure 1:**
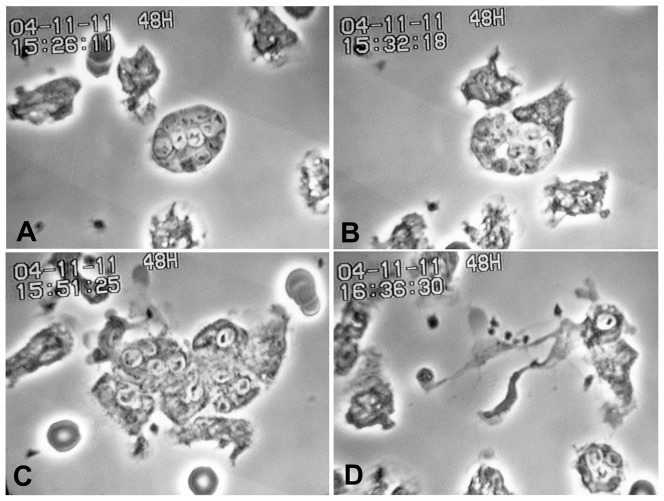
Monocyte containing zymosan attracts PMN which apparently dismantle it, taking up its zymosan themselves. A. Monocyte contains 9 zymosan. PMN approaching. B. +7 min, 7 sec: The monocyte swells (dying). The PMN continue to arrive. C. +19 min, 7 sec: PMN appear to enter the monocyte and take up its zymosan. D. +45 min, 5,sec: The monocyte is completely destroyed. Nuclear material is stretched out. PMN move away. (Monocytes and zymosan in 100% plasma, 2 hr, washed, and autologous PMN added. Substrate: methacrylate-coated glass.)

Among some 36 experiments with monocytes fed zymosan, those containing larger numbers of zymosan were more likely to be chemotactic for PMN. Swelling began either before or after the arrival of aggressor PMN. In Figure 1, followed on methacrylate-coated glass, monocytes had been given zymosan in 100% plasma for 2 hr, washed, and autologous PMN added. The attracted PMN (A) arrive and take up zymosan (B,C), and retreat with their plunder (D), leaving the monocyte in tatters. See also Video S1.

**Figure 2:**
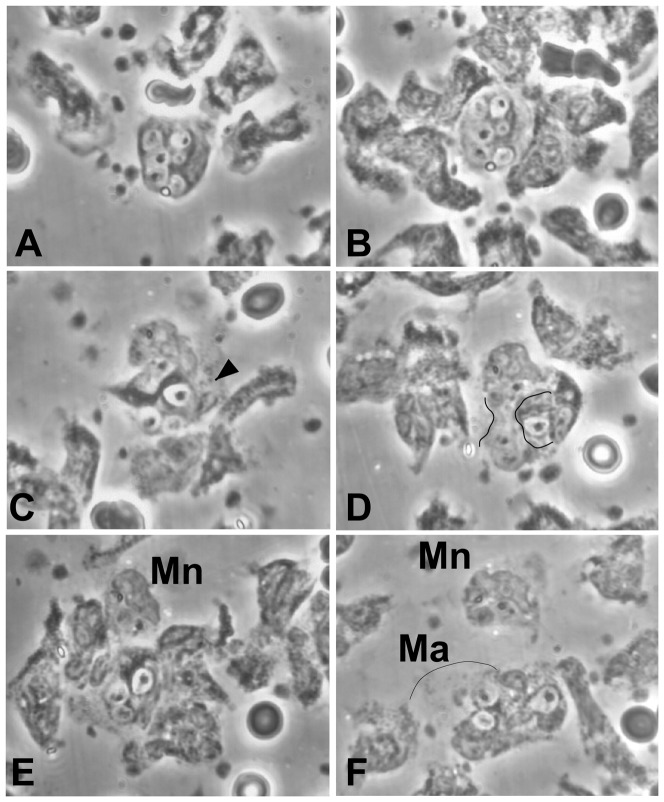
PMN can purloin a monocyte's zymosan without killing it. **A. Monocyte (M) containing 6 zymosan.** A. deformed (trapped) erythrocyte is at 12 o'clock. Four PMN are approaching. B. +6 min, 55 sec: PMN are surrounding the monocyte. C. +27 min, 43 sec: A PMN, traversing the monocyte from left to right, takes up a zymosan. D. +6 min, 56 sec: The same PMN is now pressing into the body of the monocyte from the right. The monocyte is so far intact and motile. E. +4 min,19 sec: The PMN appears to have transected the monocyte. The nucleated portion (above, Mn) remains motile. F. +15 min,58 sec: Ma indicates the anucleate portion of the monocyte. The arc shows a clear area of its residual cytoplasm; at right its zymosan is being scavenged by PMN. Mn remains motile for 24 more minutes. (Monocytes and zymosan in 100% plasma, 1 hr, washed, and autologous PMN added. Substrate: plastic.)

Figure 2 shows the rare zymosan-laden monocyte that survived the onslaught of PMN. Monocytes had been given zymosan in 100% plasma for 1 hr, washed, and autologous PMN added. Substrate was plastic. Attracted PMN appproach (A) and surround (B) the monocyte. In traversing the monocyte left-to-right, a PMN (C) takes up a zymosan. Several min later, the same PMN, now moving right-to-left (D), compresses the “waist” of the monocyte that separates its nucleated portion (above) from the portion containing most of its zymosan (below). The two portions are transected (E), their membranes apparently sealed, and the nucleated portion (Mn) moves away (F); it remains motile for at least 24 more minutes. The portion with prominant zymosan (F, Ma) is scavenged by PMN. See also Video S2.

**Figure 3:**
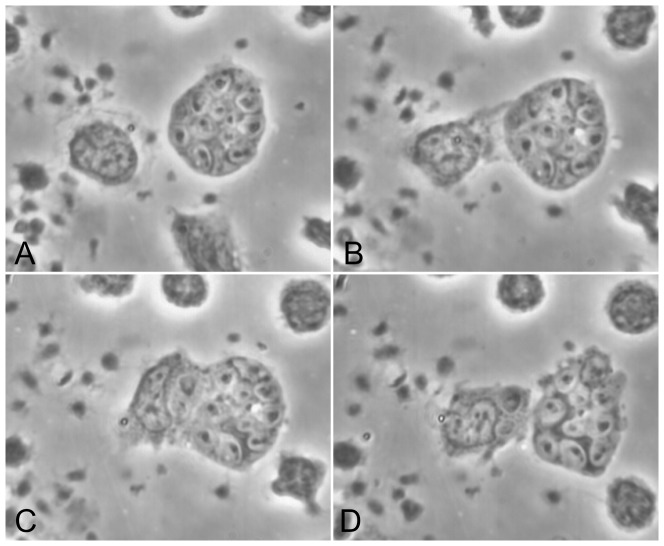
Monocytes can purloin another a monocyte's zymosan without killing it. A. Target monocyte loaded with 13 zymosan; aggressor monocyte at left. B. +11 min, 7 sec. The latter extends a wide cytoplasmic tongue, C. +5 min, 43 sec, surrounds 2 zymosan from the target cell, and D. +16 min, 34 sec, incorporates them into its own cytoplasm. (Monocytes and zymosan in 100% plasma, 2 hr, washed, and autologous monocytes added. Substrate: plastic.)

Monocytes loaded with zymosan survive better when given *monocytes* as potential aggressors (three of 5 experiments; monocytes given zymosan for 2 hr; all on plastic). In Figure 3 a target monocyte containing 11 zymosan gave up two of them to an aggressor monocyte; both monocytes remained intact. See also Video S3.

**Figure 4:**
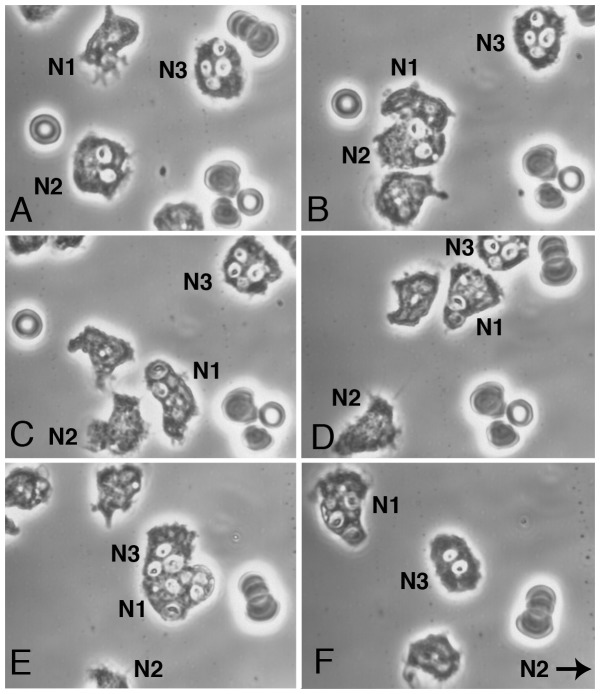
PMN can purloin zymosan from other PMN without loss of motile function in aggressor or victims. A. Three PMN (N1–3), one with 2 prominent zymosan (N2) another with 4 zymosan (N3). B +1 min, 57′sec, and C +9 min, 23 sec. Aggressor PMN (N1) takes up 2 zymosan from N2. D +3 min, 20 sec. N1 moves to N3 and, in E +7 min, 15 sec, and F 15 min, 39 sec, takes up two more zymosan from the latter. All 3 PMN remain motile (N2 has left the field at lower right). (PMN and zymosan in 100% plasma, 30 min, washed, and autologous PMN added. Substrate: glass.)

When we took to giving zymosan to PMN, a hardier cell than monocytes in slide preparations, among some 11 experiments many zymosan-laden PMN were chemotactic for other PMN, and remained intact. Aggressor PMN were capable of taking up zymosan from their victims, and they, too, tended to remain functional. In Figure 4 PMN had been given zymosan in 100% plasma for 30 min, washed, and autologous PMN added. Substrate was glass. An aggressor PMN (N1) was attracted to N2 (A), took up two zymosan from the latter (B, C), then headed for N3 (D) from which it took up another 2 zymosan (E). All three PMN remained intact. See also Video S4.

**Figure 5:**
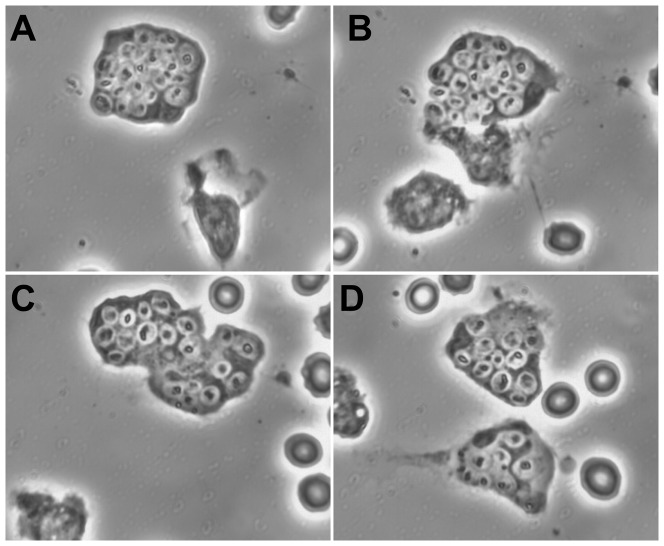
PMN can purloin zymosan from other PMN in decomplemented serum. A. A PMN approaches from below a PMN laden with 24 zymosan, B. +11 min, 36 sec, crawls about it to the left, and C. +27 min, 12 sec, removes 11 zymosan from it. D. +44 min, 4 sec. Both PMN remain motile. (PMN and zymosan in 10% decomplemented serum, washed, and autologous PMN added. Substrate: glass.)

To determine whether activated complement was essential for cellular chemotaxis we employed 10% autologous serum that had been heated at 56°C for 30 min. Among 8 experiments in which monocytes were the loaded victims, 5 experiments were negative (potential target monocytes contained 7, 5, 3, 6, or 4 zymosan). In the 3 positive experiments, where target monocytes contained 4, 5, or 6 zymosan, aggressor PMN purloined 1, 3, and 1 zymosan, respectively. Among 10 experiments in which PMN were the loaded victims, 7 experiments were negative (potential target PMN contained 3, 7, 5, 9, 5, 6, or 7 zymosan). In 2 of the 3 positive experiments, where target PMN contained 4 or 7 zymosan, aggressor PMN each purloined 1 zymosan. In an exceptional third experiment (Figure 5) an aggressor PMN approached a victim packed with over 20 zymosan and removed 11 of them. Aggressor and victim remained motile. Chemoattraction is generally weaker in decomplemented serum, but plunder is not abolished under these conditions. See also Video S5.

## Discussion

Monocytes loaded with zymosan particles can attract PMN which can ingest the monocyte's already ingested contents. Although the target monocyte rarely survives this process (Figure 1), it can occasionally do so (Figure 2). Moreover, aggressor monocytes can purloin zymosan from loaded (target) monocytes (Figure 3), and aggressor PMN from target PMN (Figure 4). Either one may do so in decomplemented serum, where chemoattraction is weaker but not globally absent (Figure 5). Both aggressors and targets may survive the exchange.

A number of questions arise. Phagocytes may extract intraerythrocytic material (Howell Jolly bodies, Heinz Bodies, malarial parasites) without destroying the red cell [1]. But so far as we know, this is the first example of nucleated cells exchanging ingested particulate material. Zymosan particles have been used for decades as surrogates for bacteria in studies of phagocytosis, so an immediate question is whether these observations can be extended to bacteria and other particles.

What chemoattractants are emanating from the target cell? Products of complement activation are not a sufficient explanation (Figure 5). Why are some neutrophils or monocytes aggressors, others, bystanders? Here, without being more specific, we can point to the emerging heterogeneity of both cell types in blood [2], [3].

Finally, if the zymosan particles are in phago(lyso)somes, particularly in PMN where both the invader and its victim may remain alive, how does the invading PMN deal with the cell membrane of the victim as well as with that of the phago(lyso)some without disrupting cellular integrity?

What follows is hypothesis, requiring ultrastuctural confirmation. It is an approach to the general problem of what and where is located the fusagen responsible for sealing cell membranes without disrupting cellular homeostasis. A clear example of such fusion is seen in a pinched human erythrocyte. Hematologists have long known that ingestion of the bi-concave red cell may produce two spherical halves, each retaining hemoglobin – i.e., membrane integrity. Similarly, in microangiopathic hemolytic anemia, the sealing of the erythrocyte membrane at folds in the cell or along lines of apposition of the membrane is thought to permit fragmentation to take place, with loss of relatively little of the contained hemoglobin [1]. In these cases it would seem that it is the inner cell membranes that, when closely opposed, fuse. We think of this spatially as an extreme hourglass configuration.

Another clear example is in the formation of cytokineplasts (CKP), motile cytoplasts from human PMN [4], [5]. When PMN are heated appropriately on surfaces, the ectoplasmic motile machinery uncouples from the endoplasm (nucleus, granules) and crawls away from it. The space between the intervening membranes narrows, and the two membranes eventually come together and fuse, freeing the CKP and leaving the residual cell body behind. Both are intact.

A more lopsided example is the migrating cell that tears away with impunity from the extreme adherent portion of its uropod [6]. Again, the retained portion of the uropod stretches until the opposing inner membranes meet and seal themselves.

This could be what we are seeing in Figure 2 where a PMN appeared to transect a monocyte, separating an intact nucleated portion (Mn) from the major zymosan-containg portion (Ma). We would suggest that inner membranes on either side of the monocyte were pushed together and fused.

**Figure 6:**
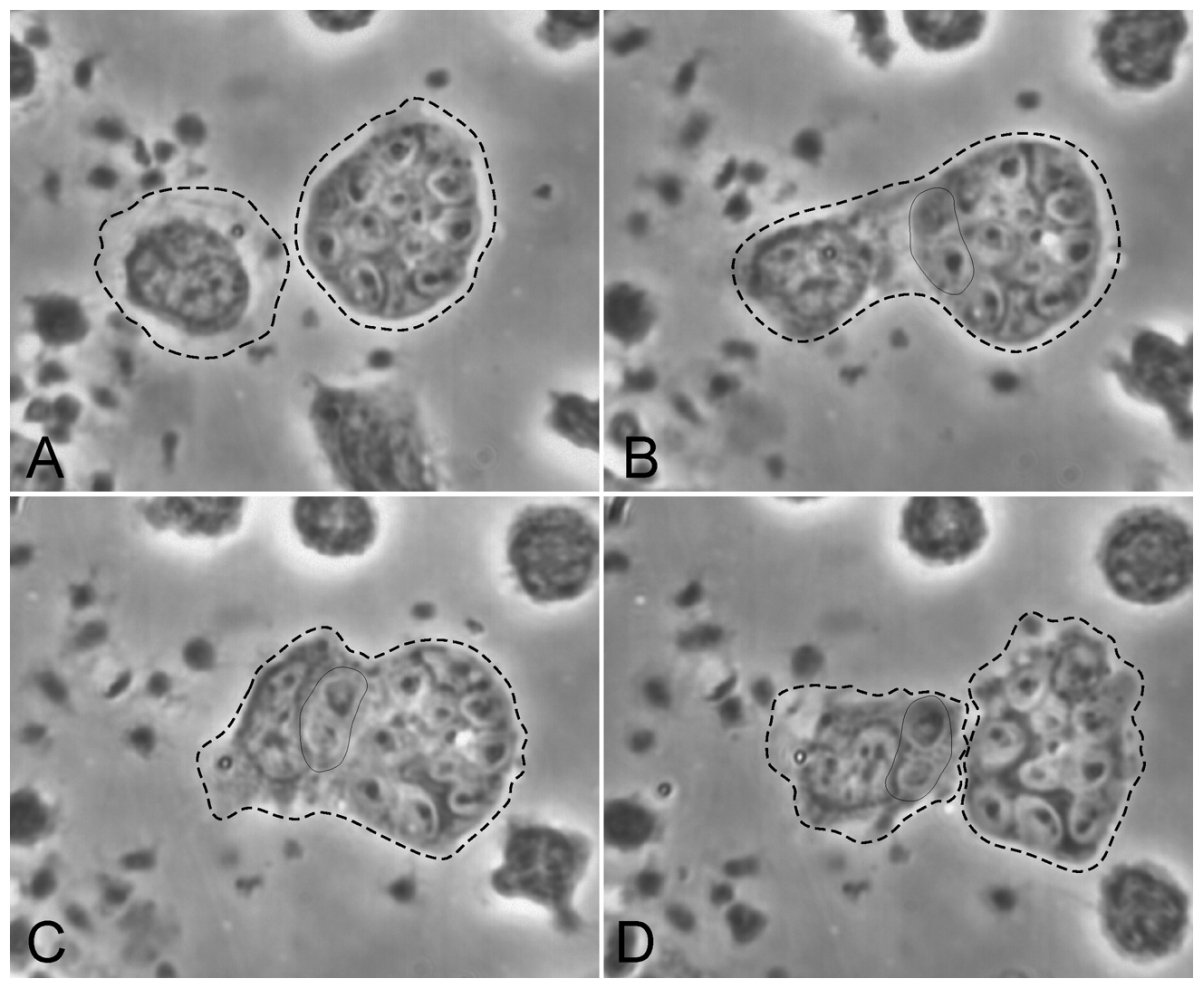
One simple interpretation of the membrane events associated with intercellular transfer of zymosan. In this formulation, the two cells briefly become one (B), a phagosome(s) moves from right to left, transporting 2 zymosan (C), and the separate cells reestablish themselves (D).

This mechanism is less obvious for explaining the removal of ingested material from one leukocyte by another, because, short of ultrastructural studies, we cannot know what membranes (cell, phagosomal) are involved. There are a number of ways in which such transfer may occur, of which one simple formulation is illustrated in Figure 6. But even for the phagocytic process in general, of which this would appear to be an example, if, invoking Occam's razor, we assume that there is only one membrane fusagen, we may then suppose its availability in intimate closing spaces. If confirmed, while not addressing what the fusagen is, this formulation might offer an explanation for where this critical agent for the maintenance of membrane integrity may reside.

## Supporting Information

Video S1 **Monocyte containing zymosan attracts PMN which apparently dismantle it, taking up its zymosan themselves.**
(MOV)

Video S2 **PMN can purloin a monocyte's zymosan without killing it. Details are best seen in the labeled portions of the still photographs.**
(MOV)

Video S3 **Monocytes can purloin another a monocyte's zymosan without killing it.**
(MOV)

Video S4 **PMN can purloin zymosan from other PMN without loss of motile function in aggressor or victims.** Details are best seen in the labeled portions of the still photographs.
(MOV)

Video S5 **PMN can purloin zymosan from other PMN in decomplemented serum.** Eleven of 24 zymosan are moved from one PMN to another; both cells remain motile.
(MOV)
